# Unexpected Difficult Intubation Reveals Congenital Tracheal Stenosis in an Adult During Anesthesia Induction for Gastroesophageal Junction Cancer Surgery

**DOI:** 10.7759/cureus.80292

**Published:** 2025-03-09

**Authors:** Atsuhiro Kitaura, Yumi Taniguchi, Haruyuki Yuasa, Hiroatsu Sakamoto, Shota Tsukimoto, Takashi Mino, Yasufumi Nakajima

**Affiliations:** 1 Anesthesiology, Kindai University Faculty of Medicine, Osaka, JPN; 2 Dental Anesthesiology, Kanagawa Dental University, Yokosuka, JPN; 3 Anesthesiology and Center for Outcomes Research, University of Texas Health Science Center, Houston, USA

**Keywords:** asthma, computed tomography, congenital tracheal stenosis, general anesthesia, tracheal intubation

## Abstract

We encountered a case of congenital tracheal stenosis (CTS) in an adult, which was unexpectedly detected during a difficult endotracheal intubation. The patient was a 75-year-old female who was admitted to our hospital for surgery for esophagogastric junction cancer. She had a 15-year history of asthma. A preoperative chest X-ray showed slight narrowing of the trachea, but at that time, a diagnosis of tracheal stenosis could not be made. General anesthesia was induced for the planned surgery, and the anesthesiologist attempted oral endotracheal intubation with a double-lumen tube. However, the attempt was unsuccessful. Narrowing of the trachea began at the level of the first rib attachment, with the tracheal rings forming a complete ring-like structure in the bronchoscope. Intubation was successfully performed using a 7-mm single-lumen tracheal tube, and anesthesia management was carried out with the use of a bronchial blocker. While most cases of CTS are diagnosed in childhood due to symptoms of airway obstruction or congenital heart disease, CTS patients with relatively mild stenosis may remain asymptomatic or undiagnosed into adulthood. Although CTS is a rare condition, it can lead to unexpected difficulty in intubation or multiple attempts of tracheal intubation, requiring caution. Preoperative evaluation, including the presence of respiratory conditions such as asthma, and the potential utility of chest X-rays and computed tomography scans for detecting CTS, were considered essential for careful preoperative assessment.

## Introduction

Congenital tracheal stenosis (CTS) is a rare condition, occurring in approximately one in 64,500 live births [1,2]. CTS is believed to result from abnormal development of the tracheal cartilage, although the exact etiology remains unclear. The definition of CTS is the presence of tracheal stenosis with a complete tracheal ring at the site of the stenosis [1-3]. CTS is frequently associated with anomalies such as bronchial branching abnormalities, congenital heart disease, and pulmonary artery sling [1-3]. In most cases, respiratory symptoms like wheezing and cyanosis manifest at around one to two months of age, often exacerbated by upper respiratory infections [1-3]. As a result, many cases are medically monitored, with some requiring surgical intervention. However, in instances of mild stenosis, airway obstruction symptoms may be minimal or absent, and many individuals remain undiagnosed until adulthood [3,4]. In these patients, CTS may first be diagnosed during endotracheal intubation performed for the management of other conditions. Unexpected difficulties in securing the airway can arise after the endotracheal tube passes through the glottis. Given the relatively low awareness of CTS among healthcare professionals in adult cases [4,5], failure to make appropriate clinical decisions may place the patient in a dangerous situation. In this report, we present a case of adult CTS that was discovered during unexpected intubation difficulties while administering general anesthesia for esophagogastric junction cancer surgery.

## Case presentation

**Figure 1:**
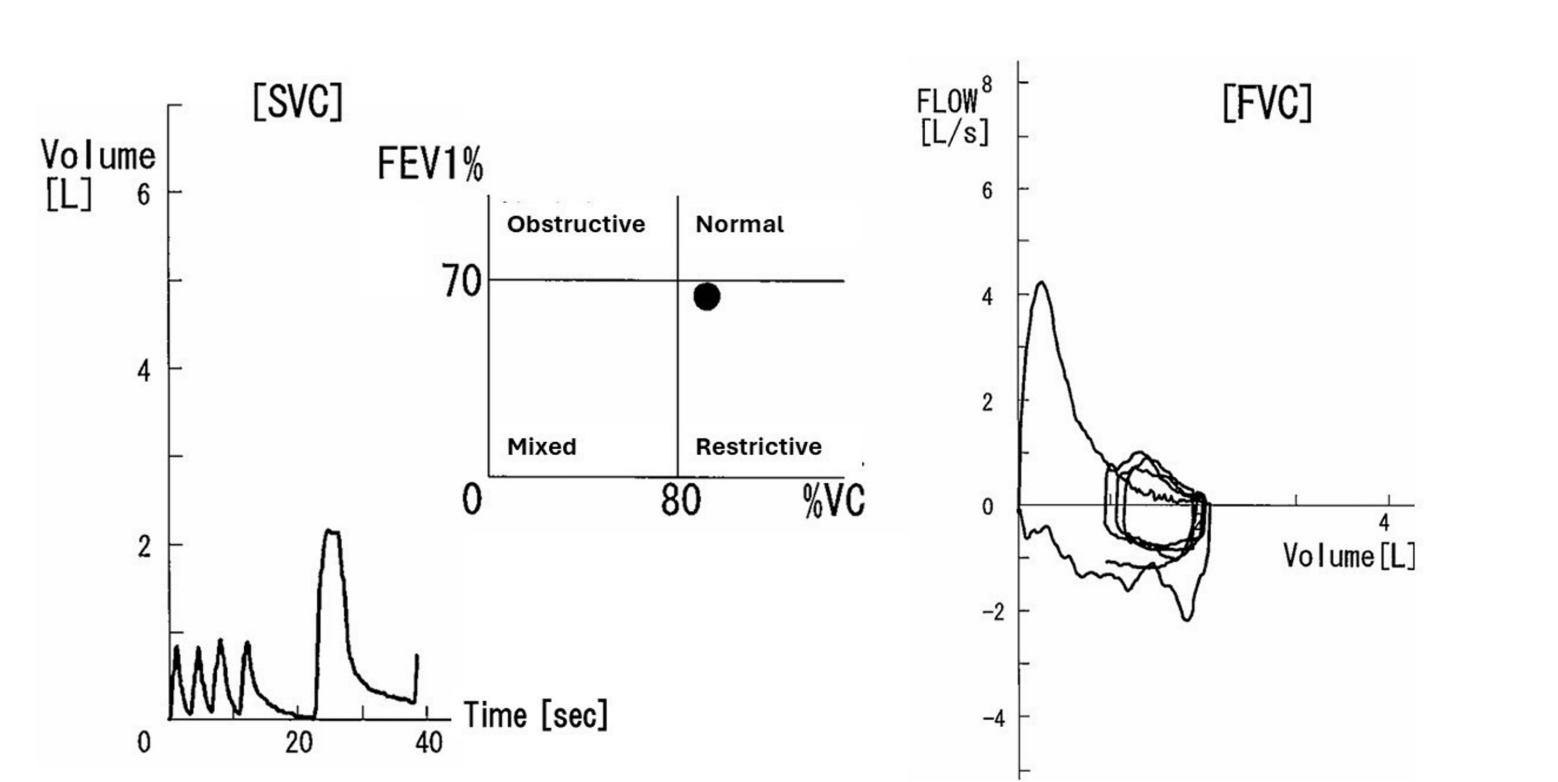
Preoperative spirometry of the present case. Mild obstructive ventilatory impairment was observed, and the flow-volume curve demonstrated a pattern consistent with bronchial asthma. FEV1: forced expiratory volume in one second; SVC: slow vital capacity; FVC: flow-volume curve; VC: vital capacity.

A 75-year-old female (height: 151 cm; weight: 73 kg) was scheduled for general anesthesia for surgery related to esophagogastric junction cancer. She initially presented to her family doctor with complaints of discomfort in the epigastric region, which led to the diagnosis of esophagogastric junction cancer, after which she was admitted to our hospital for curative surgery. Her medical history included asthma, atrial fibrillation, and diabetes. She had been diagnosed with asthma and was managed under the care of an asthma specialist for over 15 years, but no abnormalities in her trachea were detected during her previous evaluations. She was receiving treatment for asthma with inhaled corticosteroids (ICS) and inhaled long-acting β-agonists, and her asthma had been stable. Preoperative spirometry revealed that the patient exhibited mild obstructive ventilatory impairment, with no patterns of upper airway obstruction observed (Figure 1 and Table 1).

**Table 1:** Preoperative spirometry of the present case. The results of the spirometry indicated mild obstructive ventilatory impairment. FEV1: forced expiratory volume in one second; FEV1%: % of forced expiratory volume in one second.

Lung capacity	Measured value	Predicted value
Vital capacity (L)	2.15	2.32
Tidal volume (L)	0.77	-
Inspiratory capacity (L)	2.08	-
Vital capacity		
Forced vital capacity (L)	1.94	2.17
FEV1 (L)	1.24	1.68
FEV1% (%)	63.92	78.74

**Figure 2:**
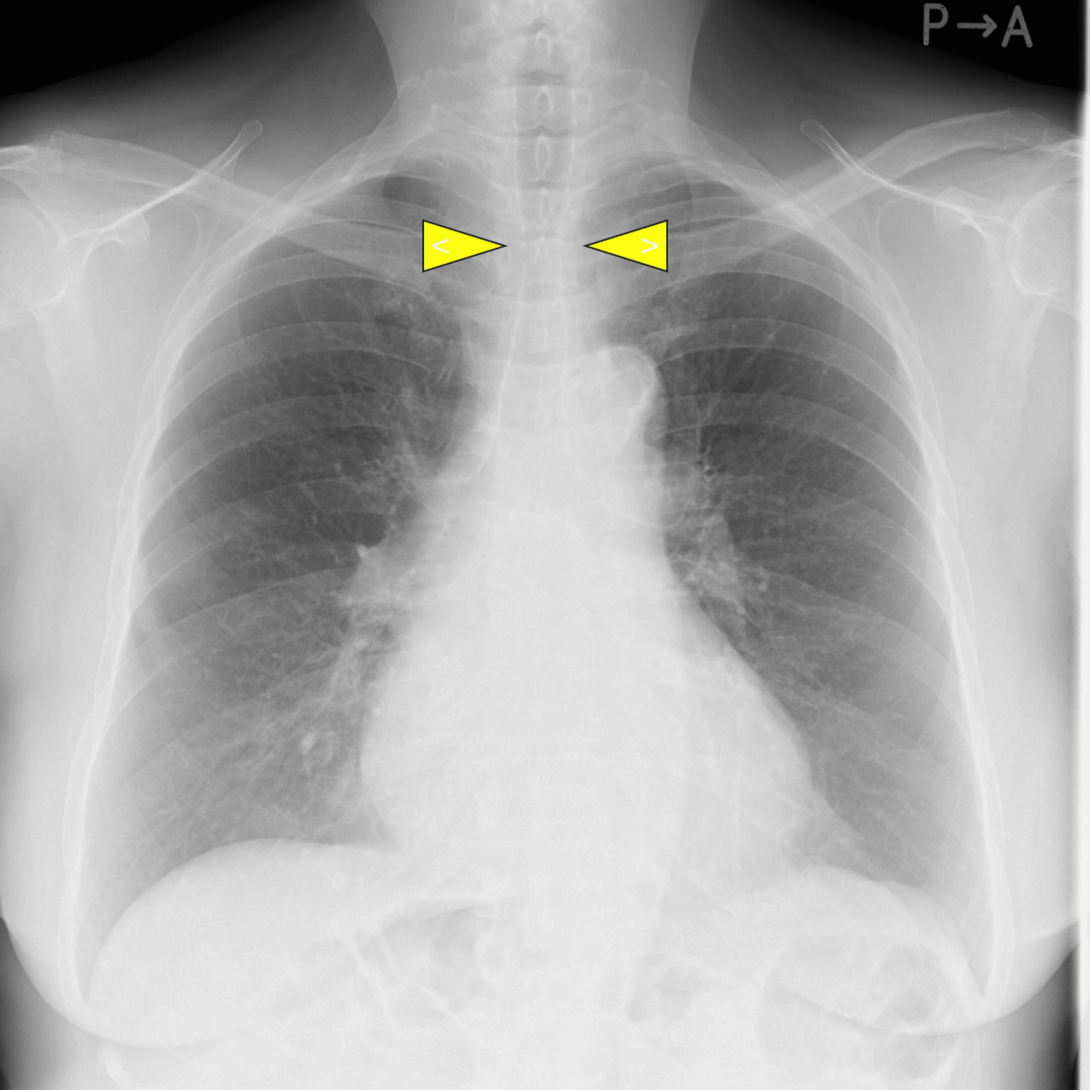
The preoperative chest X-ray image of the present case. A slight narrowing of a portion of the trachea (yellow arrows) is observed.

The planned surgery was a thoracoscopic-assisted subtotal esophagectomy with laparoscopic-assisted gastric resection and gastric tube reconstruction. A combined approach with general anesthesia and epidural anesthesia was planned. Due to the requirement for one-lung ventilation during the thoracoscopic procedure, double-lumen tube (DLT) intubation was initially planned. Preoperative chest X-rays revealed a slight narrowing of the tracheal diameter (Figure 2), but a diagnosis of CTS was not made at that time.

**Figure 3:**
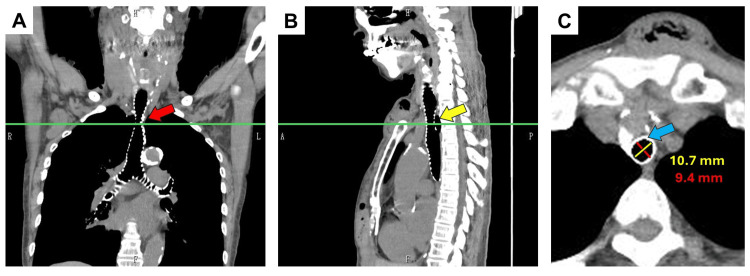
Postoperative computed tomography multi-planar reconstruction image of the present case. A: Coronal image of the trachea. Tracheal stenosis (red arrow) is observed. The green line indicates the level of the narrowest part of the trachea. B: Sagittal image. Tracheal cartilage is present on the dorsal side of the trachea (yellow arrow) at the site of the tracheal stenosis. The green line indicates the level of the narrowest part of the trachea. C: Axial image at the level of the narrowest part of the trachea. The level of the narrowest part is indicated by the green line in images A and B. A complete tracheal ring and a defect in the membranous portion are observed (blue arrow). The narrowest point measured 10.7 mm in the longitudinal diameter (yellow line) and 9.4 mm in the transverse diameter (red line).

After placement of the epidural catheter, general anesthesia was induced with propofol (1.5 mg/kg) and rocuronium (0.6 mg/kg). Upon attempting endotracheal intubation, a 32 Fr DLT (Portex Blue Line endobronchial tube, ICU Medical, Inc., San Clemente, CA; the outer diameter (OD) of the tracheal tube was 10.1 × 11.2 mm) could not be inserted. Even after switching to a 28 Fr DLT (Portex Blue Line endobronchial tube; OD of the tracheal tube was 8.8 × 9.7 mm), the tube still could not pass through the trachea. Tracheal anatomy was subsequently examined with a bronchoscope, which confirmed the presence of tracheal stenosis. Given the degree of stenosis, it was deemed impossible to use a DLT for this patient. A single-lumen tracheal tube (SLT) (Shiley^TM^ Oral/Nasal Tracheal Tube Cuffed, ID: 7 mm, OD: 9.7 mm, COVIDIEN Japan Inc., Tokyo, Japan) was smoothly inserted, and anesthesia management proceeded with an SLT and bronchial blocker, enabling the surgery to proceed successfully without further issues. After induction of anesthesia, the patient’s preoperative CT scan was re-examined, revealing five complete tracheal rings at a segment of the trachea, which accounted for the observed stenosis (Figure 3).

Tracheal stenosis was observed from the level of the first rib attachment to approximately 4 cm proximally to the tracheal bifurcation (Figures 3A, 3B). The narrowest point measured 10.7 mm in the long diameter and 9.4 mm in the short diameter (Figure 3C). No associated congenital anomalies, such as cardiac malformations or pulmonary vascular abnormalities, were identified, which are commonly associated with CTS.

Two weeks after the first surgery, the patient underwent abdominal drainage surgery under general anesthesia due to an anastomotic leak. An SLT (Shiley^TM^ Oral/Nasal Tracheal Tube Cuffed, ID: 7 mm, OD: 9.7 mm, COVIDIEN Japan Inc.) was used for intubation, and no issues arose during this procedure.

Three years after the first surgery, the patient required a second operation due to local recurrence at the anastomotic site. The planned procedure was a thoracoscopic-assisted gastric tube resection with residual esophagectomy and free ileal reconstruction under general anesthesia. Unfortunately, the anesthesiologist did not confirm the patient's previous history of difficult intubation during the first surgery. As a result, the same difficulties experienced during the first surgery were repeated during airway management. Despite these challenges, the surgery was successfully completed, and the patient was discharged 21 days postoperatively. The patient opted for postoperative chemotherapy. Unfortunately, the patient passed away three years after the second surgery due to the recurrence of esophagogastric junction cancer.

## Discussion

In the present case, CTS was detected during DLT intubation. The diagnostic criteria for CTS include the presence of tracheal stenosis on imaging studies, the identification of complete tracheal rings, the presence of airway narrowing symptoms, and the exclusion of secondary tracheal stenosis [1-3]. In the present case, the diagnosis of CTS was made based on the presence of tracheal stenosis observed in chest X-ray and CT images, the identification of typical complete tracheal rings on CT and intraoperative bronchoscopy, the presence of asthma, and the exclusion of secondary tracheal stenosis. The DLT was able to pass through the glottis but could not be advanced further into the trachea. Postoperative CT imaging revealed that the intubation difficulty was caused by a mismatch between the DLT size and the tracheal diameter at the stenotic site. Since the degree of tracheal stenosis in the present case was very mild, an SLT (ID: 7.0 mm) was successfully inserted. This size is consistent with the SLT commonly used for tracheal intubation in adult females. Therefore, it was confirmed that intubation with a standard SLT would not likely pose difficulties in this case. However, the stenotic region had only about 60% of the diameter of the normal airway, and since it coincided with the cuff placement zone, there was a higher potential risk of mucosal injury compared to usual.

The diagnosis of CTS in adults is exceedingly rare [1-3]. Our search identified a total of 20 cases in both English and Japanese literature [6-25]. Table 2 provides an overview of this case, along with others.

**Table 2:** Previously reported cases of adult congenital tracheal stenosis. F: female; M: male; CTS: congenital tracheal stenosis; CT: computed tomography; NR: not recorded; PAPVR: partial anomalous pulmonary venous return; ASD: atrial septal defect.

Case	Age/gender		The trigger for detection of CTS	Respiratory symptoms	Cardiac and pulmonary malformations	Minimum diameter of trachea (mm)	Reference
1		44/F	Chest X-ray	None	None	16	[6]
2		45/F	Difficult intubation	None	NR	<10	[7]
3		25/F	Difficult intubation	None	NR	6	[8]
4		39/F	Difficult intubation	None	NR	10	[9]
5		42/F	Difficult intubation	None	NR	6	[10]
6		53/F	Difficult intubation	None	NR	5	[11]
7		70/M	Difficult intubation	None	NR	NR	[12]
8		52/F	Difficult intubation	None	NR	8.27	[13]
9		32/F	Difficult intubation	None	NR	NR	[14]
10		29/F	Chest CT	Asthma	Anomalous origin of right upper lobe bronchus	7	[15]
11		37/F	Chest CT	Asthma	NR	6.3	[16]
12		23/F	Chest X-ray/CT	Asthma	Pulmonary artery sling, abnormal tracheal bifurcation	6.8	[17]
13		34/F	Chest CT	Shortness of breath and chest tightness	Congenital heart disease (unknown details)	8	[18]
14		42/F	Chest X-ray/CT	Dry cough	None	6	[19]
15		19/NR	NR	Asthma	NR	NR	[20]
16		60/F	Bronchoscopy	Dyspnea	NR	5	[21]
17		57/M	Chest X-ray/CT	Impaired exercise tolerance	NR	NR	[22]
18		42/F	Difficult intubation	Asthma	None	NR	[23]
19		51/F	Difficult intubation	Asthma	Pulmonary artery sling, PAPVR, ASD	6	[24]
20		21/F	Difficult intubation	Asthma	NR	7	[25]
21		75/F	Difficult intubation	Asthma	None	9.4	Current case

Of the 21 cases identified, 12, including this case, were discovered during attempts to intubate patients for surgeries related to other conditions or for pulmonary disease management [7-14,23-25]. The remaining cases were either detected incidentally or identified by chest X-ray performed to evaluate respiratory symptoms [6,15-19,22]. Only four of the 21 cases were associated with cardiopulmonary malformations [15,18,24]. Typically, 75% of cases of CTS are associated with cardiac malformations [1-3]. However, in cases of CTS that are overlooked until adulthood, the incidence of concomitant cardiac malformations tends to be low. In adult cases of CTS who are diagnosed in adulthood, it is likely that the tracheal stenosis is mild enough not to affect survival or that the condition is uncomplicated by comorbidities. As a result, these cases often go undetected in daily life.

In nine cases, including the present case, intubation was performed successfully with a smaller tracheal tube or by performing intubation proximal to the tracheal stenosis. In the remaining two cases, supraglottic devices were used [12,13]. Another case required an emergency surgical tracheostomy [10]. Anesthesiologists who encounter unexpected difficulty with tracheal intubation should consider the possibility of undiagnosed CTS.

Moreover, more than half of the reported cases had respiratory symptoms [15-25]. Long-term bronchial asthma was present in eight cases, including the present case [15-17,20,23-25]. The other four cases presented with certain respiratory symptoms [18,19,21,22]. Preoperative asthma-like symptoms may trigger suspicion of CTS. However, the incidence is extremely rare.

In the present case, CTS was not diagnosed preoperatively, preventing the anticipation of intubation difficulties. There were two opportunities to diagnose CTS preoperatively. The first was the patient’s history of bronchial asthma. Despite receiving specialized care and follow-up for asthma, CTS was not recognized prior to surgery.

The mild stenosis of the trachea (10 mm in diameter at the narrowest point), along with the absence of characteristic dyspnea during childhood and the spirometry results showing a bronchial asthma pattern (not upper airway obstruction pattern), may have contributed to the delayed diagnosis of CTS. Although rare, it is essential to consider the possibility of undiagnosed CTS in adult patients with asthma [4,5].

The second diagnostic trigger was the finding of tracheal stenosis on a preoperative chest X-ray (Figure 2). However, the degree of stenosis was not pronounced enough, and the attending anesthesiologist did not pursue further investigation. As a result of this, three attempts of intubation were done. And the patient’s risk of complications was higher than usual [26]. Upon reviewing the CT scan (Figure 3), it was clear that there are complete tracheal rings and tracheal stenosis, and the difficulty with DLT intubation could have been predicted preoperatively. This case underscores the importance of preoperative imaging and a careful review of previous anesthesia histories. To preemptively predict intubation difficulties due to adult CTS, it is crucial for healthcare providers to recognize the possibility of undiagnosed adult CTS cases. Additionally, when possible, it is important to carefully assess the airway morphology through preoperative imaging data. Furthermore, it was deemed important to establish a means of accurately sharing information about tracheal intubation issues with future healthcare providers.

## Conclusions

Unexpected difficulties in tracheal intubation may, in rare cases, be associated with CTS. Healthcare professionals' prior recognition of the potential presence of CTS, along with a careful evaluation of preoperative factors such as bronchial asthma, imaging findings, and previous anesthesia records, may aid in the diagnosis of CTS. Early diagnosis of CTS allows for the preemptive consideration of appropriate airway management techniques, thereby preventing unnecessary multiple attempts at tracheal intubation and the associated critical situations.
